# Genomic diversity and host-specificity in *Corynebacterium pseudotuberculosis* using comparative population genomics

**DOI:** 10.3389/fmicb.2026.1729846

**Published:** 2026-02-17

**Authors:** Rodrigo Profeta, Cory L. Schlesener, Claire A. Shaw, Roselle C. Busch, Meera C. Heller, Sharon Spier, Jing Wu, Shannara Welch, Marcus Vinicius C. Viana, Fernanda A. L. Barroso, Bertram Brenig, Vasco Azevedo, Bart C. Weimer

**Affiliations:** 1 Population Health and Reproduction, School of Veterinary Medicine, UC Davis, Davis, CA, United States; 2 100K Pathogen Genome Project, School of Veterinary Medicine, UC Davis, Davis, CA, United States; 3 Veterinary Medicine and Epidemiology, School of Veterinary Medicine, UC Davis, Davis, CA, United States; 4 Texas A&M Veterinary Medical Teaching Hospital, College Station, TX, United States; 5 Department of Genetics, Ecology and Evolution, Federal University of Minas Gerais, Belo Horizonte, Brazil; 6 Institute of Veterinary Medicine, University of Göttingen, Göttingen, Germany

**Keywords:** host/microbe association, machine learning, pangenome, point mutation, SNP

## Abstract

Corynebacterium *pseudotuberculosis* is a facultative intracellular pathogen responsible for chronic infections in livestock, primarily small ruminants and horses, with occasional zoonotic transmission. To investigate the genomic diversity, evolutionary stability, and host adaptation of this species, we analyzed 788 high-quality genomes representing isolates from diverse hosts, geographic regions, and time periods. Comparative population genomics revealed remarkably conserved genome architecture, supporting a closed pangenome with minimal accessory gene variation. Virulence and antimicrobial resistance (AMR) screening across multiple databases confirmed the universal presence of phospholipase D (pld) and the absence of major horizontally acquired AMR determinants, except for APH(3’)-IIa, TEM-116, and APH(3’)-IIIa in a few goat isolates from Brazil. Distinct metabolic features between biovars were conserved, notably nitrate reduction and molybdenum cofactor biosynthesis in biovar equi. However, gene presence/absence alone did not explain host specificity. Instead, machine learning applied to 8,028 core-genome SNPs identified allelic variants associated with host origin, particularly in genes linked to amino-acid biosynthesis and peptide transport (Opp system). These findings demonstrate that host adaptation in this species is driven by fine-scale SNP variation within core metabolic pathways, rather than acquisition of classical virulence or resistance genes, highlighting the species’ exceptional genomic stability and narrow evolutionary flexibility.

## Introduction

*Corynebacterium pseudotuberculosis* is a facultative intracellular bacterial pathogen affecting primarily livestock, small ruminants, and equids. Infections caused by this organism are an increasing concern, especially in animal production where outbreaks lead to severe economic losses. These occur due to the need for culling, condemnation of whole carcasses at slaughter, and deterioration in body condition that reduces the value of other animal products such as pelts and leather (Dorella et al., 2006; O’Hara et al., 2021; Esmaeili et al., 2025). Transmission occurs through skin abrasions or mucous membranes, with soil and insect vectors acting as major reservoirs and vehicles for infection (Baird and Fontaine, 2007). In addition, sheep-to-sheep transmission via respiratory lesions has been recognized as a significant factor in the spread of disease within flocks (Ellis et al., 1987). While infections have historically been associated primarily with summer months in arid regions of the western United States, reports suggest this pathogen has undergone a geographical expansion over the past two decades (Spier, 2008). Environmental persistence and potential climate-driven changes in vector populations are believed to contribute to this rising prevalence and extended geographic reach of the disease (Spier et al., 2012). Though a pertinent concern in veterinary medicine, knowledge of the epidemiology and pathogenesis of *C. pseudotuberculosis* is incomplete, allowing the species to become endemic in some regions (Dorella et al., 2006). This limited understanding is partly due to the lack of specific diagnostic tools and the historically low-resolution methods used to differentiate strains. Although genomic studies have revealed relatively low diversity within *C. pseudotuberculosis* populations (Soares et al., 2013), the scale and depth of these analyses remain limited. As a result, important questions continue regarding the genomic basis of the organism’s resilience, persistence, adaptability, and control in multiple hosts and environments.

This organism has two major biovars that are genetically and metabolically distinct; biovar ovis, which typically infects small ruminants such as sheep and goats, and biovar equi, which primarily infects horses and occasionally cattle (Biberstein et al., 1971). Although cross-species transmission between horses and small ruminants is rare, cattle have been documented to harbor strains from either biovar (Hiller et al., 2024). In conjunction with host specificity, the major diagnostic differentiation between biovars ovis and equi is limited to their ability to reduce nitrate, with equi strains being nitrate-positive and ovis being nitrate-negative (Dorella et al., 2006).

In horses, *C. pseudotuberculosis* biovar equi infection has three clinical presentations: external abscesses, internal organ infection, or ulcerative lymphangitis. The most common manifestation involves subcutaneous abscesses, especially in the pectoral region or ventral abdomen, often referred to as “pigeon fever” due to the characteristic swelling. While external infections are generally self-limiting, internal abscesses and lymphangitis are more severe and can require prolonged antibiotic treatment (Pratt et al., 2005). Small ruminant infections share a similar presentation, with *C. pseudotuberculosis* biovar ovis infection resulting in notable abscess development both externally and internally (Baird and Fontaine, 2007).

Despite the agricultural and economic importance of *C. pseudotuberculosis*, in depth comprehensive genomic analyses have been limited, hindering the ability to make informed decisions about how best to control infections based on strain variation and presentation symptoms. The advent of whole genome sequencing (WGS) and population-scale comparative genomics has revolutionized our ability to characterize understudied pathogens. When integrated with machine learning, these data-rich methods enable the identification of subtle genomic signatures associated with host specificity, virulence, and antimicrobial resistance, offering new insights that traditional analyses may overlook (Bandoy and Weimer, 2020). However, most studies of *C. pseudotuberculosis* have relied on small isolate collections or targeted traits, resulting in a fragmented understanding of the species genomic landscape and what role genome variation plays in disease manifestation (Soares et al., 2013). Consequently, the full extent of its genomic diversity, including genes associated with host adaptation, virulence, and resistance, remains poorly characterized.

In this study, we sequenced 571 genomes and leveraged publicly available *C. pseudotuberculosis* genomes to conduct a population genome comparison. This included integrating detailed metadata including host species, geographic origin, isolation date, and antimicrobial treatment history into the analyses of genetic variance across isolates. While this comparison confirmed the known differences between biovars ovis and equi, it found that gene presence/absence did not play a role in disease characteristics or animal host. Rather, we discovered single nucleotide polymorphisms (SNP) in many core genes contributed to the observed epidemiological variations. This study uncovered the role of allelic variants in genes important in protein metabolism in variations for host association and disease presentations for both biovars.

## Materials and methods

### Genomic data collection

A total of 788 *Corynebacterium pseudotuberculosis* genome sequences were analyzed in this study. Genomes were obtained from multiple sources, including publicly available sequences from GenBank (*n* = 233), a research isolate collection at UC Davis dating back to 1996 (Rhodes et al., 2015), and clinical isolates recovered at the UC Davis Veterinary Medical Teaching Hospital up to 2021. Additional isolates were collected at Texas A&M University between 2010 and 2017, bringing the combined total from UC Davis and Texas A&M to 380. The remaining genomes were obtained from collections in Brazil. Comprehensive metadata for all isolates is provided in Supplementary Table 1.

### gDNA extraction

For UC Davis isolate collection, isolate frozen stocks (80°C) were used to inoculate vented culture tubes with 5 mL of tryptic soy broth, or brain heart infusion broth (BD Difco, Franklin Lakes, NJ, United States) supplemented with 0.2% (v/v) polysorbate 80. Cultures were incubated aerobically with shaking at 37°C for approximately 48 h. Bacteria were pelleted in microcentrifuge tubes by centrifugation at 16,000×g for 5 min, resuspended in phosphate buffered saline to wash, and pelleted again. The supernatant was removed, and pellets were stored at –80°C until DNA was extracted. DNA extraction was done with the Wizard Genomic DNA Purification kit (Promega, Madison, WI, United States). Cell pellets were lysed by suspending in Nuclei Lysis Solution from the kit, addition of 0.1 mm Mini-BeadBeater glass beads (BioSpec Products, Bartlesville, OK, United States), and bead beat for 15 s at 16,000 RPM on FastPrep-96 platform (MP Biomedicals, Santa Ana, CA, United States). Cell lysates were further processed following kit procedure. The DNA pellets were rehydrated in 100 μL of 10 mM Tris-HCl (pH 8.0). Genomic DNA quality was evaluated as described previously (Jeannotte et al., 2014; Kong et al., 2014). Briefly, purity for protein and organic contamination was assessed by Nanodrop One UV-Vis Spectrophotometer (ThermoScientific, Waltham, MA, United States), using the thresholds A260/280 ≥1.5 and A260/230 ≥1.5 for accepted samples. Genomic DNA integrity was evaluated by genomic DNA TapeStation (Agilent 4200, Santa Clara, CA, United States). The DNA was stored at –20°C until processed for Whole genome sequencing.

For the Brazilian isolate collection, genomic DNA was extracted using the Wizard^®^ Genomic DNA Purification Kit (Promega), according to the manufacturer’s instructions, as previously described (Sousa et al., 2025). DNA quantity and purity were assessed spectrophotometrically (260/280 ratio; NanoDrop 2000, Thermo Fisher Scientific) and verified by agarose gel electrophoresis. DNA quality was routinely assessed by spectrophotometric purity (260/280 ratio) and agarose gel electrophoresis. Concentrations were standardized across samples, and approximately 5 μg of high-quality DNA per isolate was prepared and submitted for sequencing.

### Library prep and sequencing

Whole genome sequencing followed methods previously described for studies under the 100K Pathogen Genome Project (Weis et al., 2016; Chen et al., 2017; Weimer, 2017). Briefly, high-quality genomic DNA was used to construct sequencing libraries with 400–550 bp inserts by enzymatic sheering, followed by size selection to an average of 450 bp and sequenced to target 50x depth of coverage per genome. Sequencing was done using paired-end 150 short read method of the Illumina HiSeq X platform (San Diego, CA, United States). Raw sequence information is available on NCBI database under the 100K Pathogen Genome Bioproject (PRJNA203445).

### Genome assembly

Raw paired-end reads were trimmed to remove adapters and low-quality bases using Trimmomatic (v0.39) (Bolger et al., 2014) with the following parameters: ILLUMINACLIP:<adapters>:2:40:15, LEADING:2, TRAILING:2, SLIDINGWINDOW:4:15, and MINLEN:50. Read quality was assessed before and after trimming using FastQC (v0.11.9) (Andrews, 2010), and summary reports were compiled using MultiQC (v1.21) (Ewels et al., 2016). Potential PhiX or other sequencing contaminants were removed by aligning reads to a PhiX reference genome using Bowtie2 (v2.5.1) (Langmead and Salzberg, 2012), retaining only unmapped read pairs. *De novo* genome assemblies were generated using Shovill (v1.0.4) (Seemann, 2022) with default parameters and the SPAdes assembler. Assembly quality was assessed with CheckM2 (v1.0.1) (Chklovski et al., 2023), and sequencing depth was estimated using Mosdepth (v0.3.8) (Pedersen and Quinlan, 2018). Assemblies were considered high quality if they met the following criteria: sequencing coverage > 10×, estimated genome completeness above 95%, and contamination below 5%.

### Genomic similarity comparison

Genomic comparisons were conducted using Sourmash (v4.8.5) (Brown and Irber, 2016). For each genome, MinHash signatures were computed using scaled sketches (–scaled 10), and pairwise Jaccard distances (*k* = 31) were calculated and exported to a distance matrix. To visualize genome-wide similarity patterns, we used ComplexHeatmap (v2.21.1) (Gu et al., 2016) in R to generate a heatmap from the distance matrix, including sample annotations indicating the host and geographical locations of the genomes.

### Pangenome analysis

All genome assemblies were annotated using Prokka (v1.14.6) (Seemann, 2014) with default parameters. The annotated GFF files were then used as input for pangenome construction. Pangenome analysis was conducted using Roary (v3.13.0) (Page et al., 2015), which clusters orthologous genes across annotated genomes based on amino acid similarity (≥95% similarity). Separate analyses were performed for *C. pseudotuberculosis* biovars ovis and equi, followed by a combined analysis of all genomes. Roary was executed with the –mafft option to generate multiple sequence alignments of core genes.

### Gene discovery

The gene presence/absence matrix generated by Roary was used to investigate the gene discovery dynamics of the pangenome. We performed rarefaction analysis in R (v4.2.3) using the micropan (Snipen and Liland, 2015) package to evaluate how the number of unique genes increases as more genomes are sampled. For each of 100 bootstrap replicates, genomes were incrementally subsampled from one up to the total number of genomes (N). At each sampling step, the cumulative count of unique, non-redundant genes was recorded. The average number of genes across all replicates was then plotted against the number of genomes on a log–log scale to generate rarefaction curves according to Heaps’ law, expressed as:


$$G\,\left(n\right)=k\cdot n^{b}$$

where *G(n)* represents the number of unique genes observed in *n* genomes, *k* is a constant, and *b* is the gene discovery rate exponent. The model provided estimates of the exponent (*b*), its standard error (*SE*), and the coefficient of determination (*R*^2^), which were used to evaluate model fit and gene discovery dynamics. For comparative purposes, we applied the same rarefaction analysis and Heaps’ law modeling pipeline to estimate the exponent *b* for additional species, including *Corynebacterium glutamicum*, *Mycobacterium tuberculosis*, and *Helicobacter pylori*. Genomes were downloaded from GenBank and subjected to the same analysis. Results from other species (*Lactococcus lactis*, *Pasteurella multocida*, *Mannheimia haemolytica*, *Escherichia coli*, *Salmonella* spp., and *Campylobacter* spp.) were included from previous studies (Kaufman et al., 2020; Garzon et al., 2025), and are summarized in Table 1.

**TABLE 1:** Genomic characteristics and gene discovery dynamics for Corynebacterium pseudotuberculosis and selected bacterial species.

Organism	Gram status	Number of genomes compared	Genome size (Mb)	GC content (%)	Number of CDSs	Gene discovery rate (b ± SE)	Genomes per new gene (1/b)	References
*Corynebacterium pseudotuberculosis* (camelid)	Positive	22	2.3	∼52%	2,035	0.037 ± 0.00166	26.70	This work
*Corynebacterium pseudotuberculosis* (ovis)	Positive	380	2.3	∼52%	2,035	0.065 ± 0.00077	15.29	This work
*Corynebacterium pseudotuberculosis*	Positive	788	2.3	∼52%	2,035	0.1005 ± 0.0006	10	This work
*Corynebacterium pseudotuberculosis* (equi)	Positive	385	2.3	∼52%	2,035	0.101 ± 0.00132	9.90	This work
*Corynebacterium diphtheriae*	Positive	435	2.5	∼54%	2,267	0.225 ± 0.00016	4.44	This work
*Corynebacterium glutamicum*	Positive	76	3.3	∼54%	3,000	0.235 ± 0.00144	4.26	This work
*Mycobacterium tuberculosis*	Positive	4,900	4.4	∼65%	4,024	0.237 ± 0.00032	4.22	This work
*Pasteurella multocida*	Negative	1,194	2.3	∼40%	2,029	0.321 ± 0.00042	3.12	(Garzon et al., 2025)
*Mannheimia haemolytica*	Negative	2,418	2.5	∼41%	2,589	0.326 ± 0.001	3.07	(Garzon et al., 2025)
*Helicobacter pylori*	Negative	3,741	1.6	∼39%	1,520	0.454 ± 0.00052	2.20	This work
*Escherichia coli*	Negative	3,300	4.6	∼51%	4,377	0.462 ± 0.002	2.16	(Kaufman et al., 2020)
*Salmonella* spp.	Negative	792	4.8	∼52%	4,600	0.468 ± 0.001	2.14	(Kaufman et al., 2020)
*Lactococcus lactis*	Positive	702	2.5	∼32%	2,310	0.479 ± 0.000063	2.59	Unpublished data
*Campylobacter* spp.	Negative	17,000	1.6	∼30%	1,654	0.645 ± 0.001	1.55	(Kaufman et al., 2020)

Genomic characteristics of the organisms being compared are shown, such as Gram Status, Genomes size, GC content, and Number of CDSs. “Gene Discovery Rate (b ± SE)” reflects the slope of the log-log regression between the number of genomes and the cumulative number of genes. “Genomes per New Gene (1/b)” estimates the number of additional genomes needed to discover one new gene.

### Allelic variant extraction and sequence comparison of the gene *fatD*

To investigate allelic variants of the biovar-specific petrobactin import system permease protein (*fatD*) from two distinct gene clusters in the Roary pangenome analysis—one exclusive to biovar equi and the other to ovis—we performed a sequence comparison. First, we extracted the corresponding locus tags for each genome from the gene_presence_absence.csv file generated by Roary (Page et al., 2015). Using the annotated gene coordinates from Prokka-generated GFF files (Seemann, 2014), we determined the contig, start, and end positions of each *fatD* homolog. These coordinates were formatted into a BED file and used to extract nucleotide sequences from the genome assemblies (.fna files) with SeqKit (Shen et al., 2016) via the subseq command: seqkit subseq –bed “$BED_FILE” “$FNA” > “$OUT_FASTA.” For each biovar, we created multiple sequence alignments of the extracted *fatD* sequences using MAFFT (Katoh and Standley, 2013), followed by consensus sequence generation with the cons command from the EMBOSS suite (Rice et al., 2000): cons -sequence ${BIOVAR}_fatD_aligned.fna -outseq ${BIOVAR}_consensus.fna.

Consensus sequences were then aligned using BLASTn to assess allelic variation. Although six nucleotide differences were identified between the ovis and equi *fatD* alleles, both translated into identical amino acid sequences, suggesting functional conservation across biovars.

### Virulence and resistance screening

To identify *in silico* virulence and antimicrobial resistance (AMR) genes, all genome assemblies were screened using ABRicate (v1.0.1) (Seemann, 2020). For virulence profiling, the VFDB was queried. For resistance screening, assemblies were compared against the CARD, NCBI AMRFinderPlus, ResFinder, ARG-ANNOT, and MEGARes databases. ABRicate was executed with default parameters for each database, and gene presence was summarized across all genomes using the –summary function.

### Variant calling and core genome alignment

Variant calling was performed using Snippy (v4.6.0) (Seemann, 2015). For biovar ovis isolates, sequencing reads were aligned to the *C. pseudotuberculosis* strain 1002B genome (Accession: GCF_001433475.1), whereas biovar equi isolates were aligned to strain 258 (Accession: GCF_000263755.5). Each sample was processed individually. Resulting outputs were merged with snippy-core to obtain a full alignment. Low-quality and gappy alignment regions were removed using snippy-clean_full_aln, and recombination events were detected and masked using Gubbins (v2.3.4) (Croucher et al., 2015). The recombination-filtered alignment was then reduced to core SNPs using SNP-sites (v2.5.1) (Page et al., 2016). A phylogenetic tree was constructed using FastTree (v2.1.11) (Price et al., 2009) under the GTR model with nucleotide input. The final tree was visualized and annotated using the Interactive Tree of Life (iTOL) (Letunic and Bork, 2021).

### SNP-based host prediction using machine learning

To identify single-nucleotide polymorphisms (SNPs) associated with host specificity in *C. pseudotuberculosis*, machine learning models were developed using the XGBoost algorithm (v1.7.8.1) (Chen and Guestrin, 2016). SNP presence/absence data were formatted as binary matrices, with each dataset annotated according to host classification. Models were implemented in R using the XGBoost, caret, and dplyr packages. Multi-class classification was performed using a softmax objective (multi:softprob), with model parameters set to eta = 0.3, max_depth = 6, and nrounds = 100, incorporating early stopping to prevent overfitting. Each dataset was partitioned into training (80%) and testing (20%) subsets using stratified sampling to preserve class proportions. Model performance was evaluated using confusion matrices and classification accuracy on the held-out test data. Feature importance scores were extracted from the trained models using built-in XGBoost methods. SNPs with high importance scores were prioritized as potential markers of host specificity. The resulting ranked SNPs were compared to findings from pangenome and core-genome SNP analyses to identify convergent genomic signatures linked to ecological adaptation.

### Functional enrichment and network analysis

To investigate the biological relevance of top-ranked SNPs associated with host species classification, functional annotation and pathway analysis were performed. Genes linked to high-importance SNPs identified by XGBoost were mapped to metabolic and regulatory pathways using BioCyc Pathway Tools (Karp et al., 2018). In parallel, protein-protein interaction networks were reconstructed using the genes of the top-ranked SNPs in the STRING database (v11.5) (Szklarczyk et al., 2021), enabling the identification of enriched functional modules.

## Results

### Population structure analysis

**FIGURE 1:**
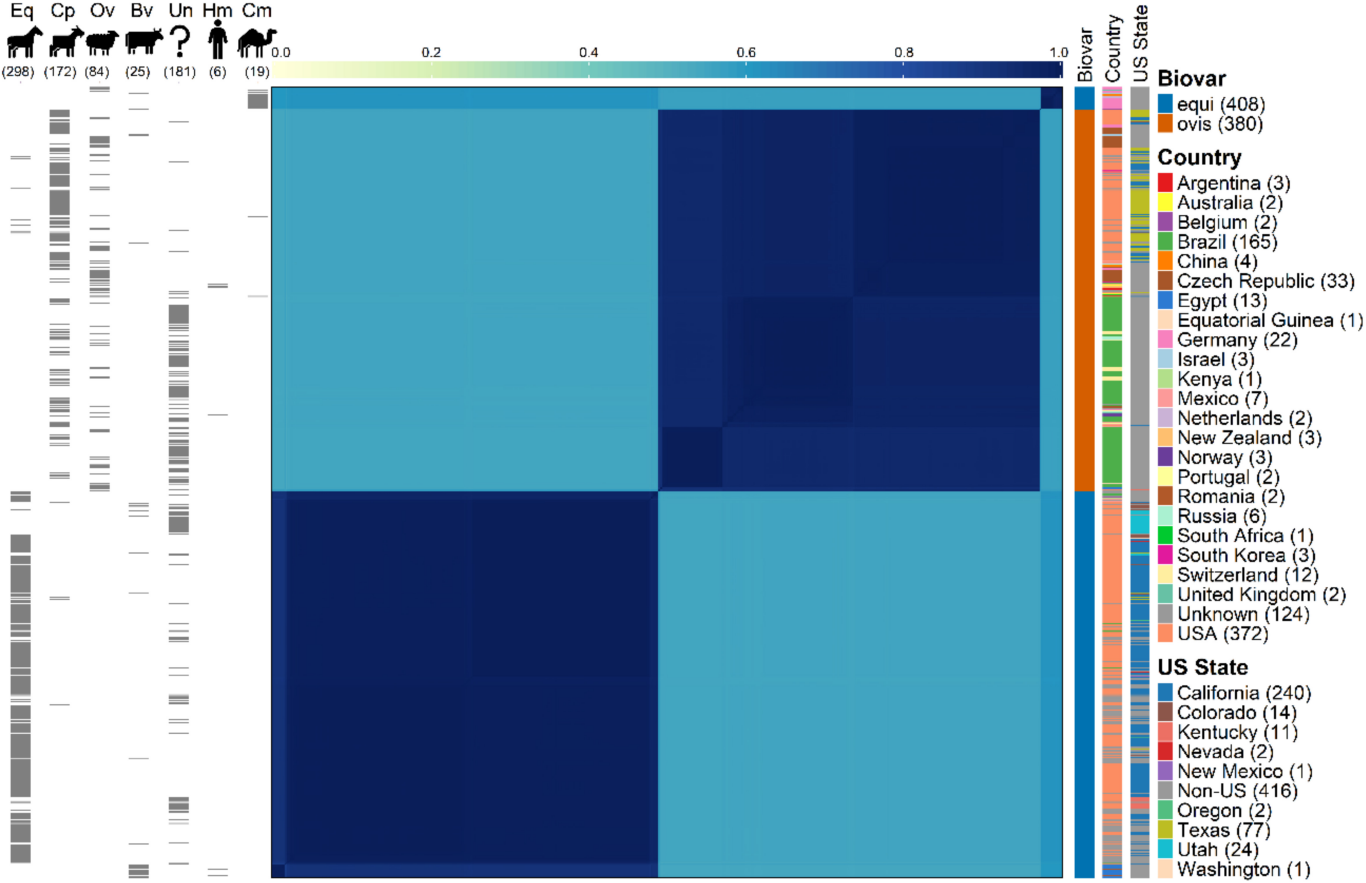
Pairwise genomic similarity among 788 *Corynebacterium pseudotuberculosis* isolates. The heatmap shows Jaccard similarity indices across genomes from 788 *C. pseudotuberculosis* strains, grouped into biovars “Equi” (408 isolates) and “Ovis” (380 isolates). Darker shades indicate higher genetic similarity. Clustering shows two large, biovar-specific blocks corresponding to the canonical biovars equi (385 isolates) and ovis (380 isolates). A third, smaller cluster—composed primarily of camelid-derived equi strains (17/22) — highlights a distinct gene-content profile within this group. Gray tick marks to the left annotate host species: Eq (equine, 298 isolates), Cp (caprine, 172), Ov (ovine, 84), Bv (bovine, 25), Un (unknown, 181), Hm (human, 6), and Cm (camelid, 19). Three singletons (one feline, one cervid and one wildebeest isolate) are omitted from this left annotation panel for clarity. Colored vertical bars to the right provide (i) biovar classification (blue, equi; orange, ovis), (ii) country of origin (23 categories plus “unknown”), and (iii) U.S. state for American isolates (California, Colorado, Kentucky, Nevada, New Mexico, Oregon, Texas, Utah, and Washington; gray marks represent non-U.S. isolates).

A total of 788 good-quality genome assemblies of *C. pseudotuberculosis* were assessed in this study, comprising 408 isolates classified as biovar equi and 380 isolates classified as biovar ovis. K-mer–based genomic similarity profiling, using a sketch size of 10, enabled fine-scale resolution of whole-genome comparisons and revealed patterns consistent with the underlying population structure of the species. The genomes clustered into three distinct genomic groups: two corresponding to the classical ovis and equi biovars, and a third novel equi cluster composed predominantly of camelid-derived isolates (Figure 1). Sequence conservation within each genomic cluster was remarkably high, as reflected by pairwise Jaccard similarity values: 0.868–1 for the equi cluster, 0.888–1 for the ovis cluster, and 0.970–1 among camelid-associated strains. The narrow range of pairwise Jaccard similarity values indicates high genomic homogeneity within each cluster. The majority of biovar ovis isolates were associated with small ruminants, while most biovar equi isolates originated from equine samples, consistent with historical reports of host specificity for these biovars (Costa et al., 1998; Haas et al., 2017; do Nascimento Sousa et al., 2024). A total of 25 bovine-derived isolates were identified in the dataset. Of these, 22 were assigned to biovar equi and 3 to biovar ovis. 1 assigned to the biovar equi cluster with the camelid cluster. This supports previous observations that cattle can act as incidental hosts for both biovars, and have strains similar to all genome types (Almeida et al., 2017). While most genomes were consistent with expected biovar—host associations based on epidemiological reports, several exceptions indicated instances of cross-host infection. Specifically, seven ovis biovar genomes were isolated from equine hosts, and eight equi biovar genomes were obtained from small ruminants (four from caprine and four from ovine sources). Also notable in this comparison was the inclusion of six human-derived isolates: two equi strains from Romania and four ovis strains, including three from New Zealand and one from Oslo.

While there was distinct biovar-level genomic clustering and expected species-association, no meaningful population structure was observed related to geographic origin. Limited clustering was observed by country or US state, however, a high level of genomic similarity by Jaccard index (>0.87) was observed. The origins of the isolates included globally diverse locations such as Brazil, Germany, New Zealand, Romania, Switzerland and the United States. The lack of distinct subclusters related to geographic location coupled to the monolithic genome structure indicates that an unusually highly conserved genomic gene content exists for each biovar globally.

### Limited gene discovery suggests a closed pangenome in *C. pseudotuberculosis*

To quantify the relationship between genome sampling and gene diversity, we applied a rarefaction analysis based on Heaps’ law, as described in the methods. This model characterizes how the number of unique genes grows with increasing genome sampling, with the Heaps’ exponent (*b*) serving as a metric of pangenome openness. Higher values indicate more dynamic and diverse gene repertoires, while lower values suggest greater genomic conservation. As previously observed (Tettelin et al., 2005) the discovery of new genes follows a power law model (log-log scale), where an increase in the number of genomes sequenced leads to a proportional increase in the number of genes discovered. The overall gene discovery rate for *C. pseudotuberculosis* was remarkably low, with a fitted exponent of *b* = 0.1005. This implies that approximately 10 genomes are required to identify one additional unique gene (1/b ≅ 10), reflecting a highly conserved gene repertoire despite extensive sampling (*n* = 788). Subgroup analyses within *C. pseudotuberculosis* revealed subtle but informative differences. The equi biovar cluster (*n* = 385) showed a comparable exponent (*b* = 0.101), while the ovis biovar (*n* = 380) was slightly more conserved (*b* = 0.065), requiring over 15 genomes to discover one new gene. The most conserved subgroup was the camelid-associated cluster (*n* = 22), with a Heaps’ exponent of just *b* = 0.037, indicating that nearly 27 genomes would be needed per novel gene identified. These values collectively underscore the exceptional genomic stability of *C. pseudotuberculosis*, particularly in host-associated subgroups. This genomic stability points to an evolutionary path that is highly constrained, reflecting long-term adaptation to specific hosts and ecological niches. Rather than gaining or losing genes frequently, *C. pseudotuberculosis* appears to have refined existing genetic functions to optimize survival within its preferred environments. The closed nature of its pangenome supports the view of limited horizontal gene transfer, strong purifying selection, and a largely clonal population framework. Consequently, most diversification within the species arises from subtle sequence-level mutations and metabolic fine-tuning instead of major shifts in gene content. Together, these features suggest that the bacterium’s specialized pathogenic way of life depends on a stable and well-conserved genetic toolkit that ensures effective colonization, persistence, and transmission in its hosts.

In contrast, other pathogens exhibited substantially more open pangenomes. Close relatives such as *Corynebacterium diphtheriae* (*b* = 0.225) and *C. glutamicum* (*b* = 0.235) demonstrated higher gene discovery rates, with only ∼4 genomes required per new gene. *Mycobacterium tuberculosis*, despite its known clonal structure, exhibited a comparable exponent (*b* = 0.237), suggesting greater gene content variation. Among Gram-negative pathogens of veterinary importance, *Pasteurella multocida* (*b* = 0.321) and *Mannheimia haemolytica* (*b* = 0.326) showed even higher pangenome openness (Garzon et al., 2025), requiring ∼3 genomes per novel gene. Further comparisons with additional species such as *Helicobacter pylori*, *Escherichia coli*, *Salmonella* spp., *Lactococcus* lactis, and *Campylobacter* spp.— which ranged from *b* = 0.454 to 0.645— highlighting the contrast in pangenome dynamics. These organisms typically required only 1.5–2.2 genomes to yield a new gene, consistent with highly open pangenomes. To evaluate whether gene discovery rates differ between these Gram-positive and Gram-negative bacteria, we compared their mean discovery rates using the Kruskal—Wallis rank sum test. This non-parametric test yielded a chi-squared statistic of χ^2^ = 3.33 (*p* = 0.068), indicating a nonsignificant trend toward higher gene discovery rates in Gram-negative species. Although this result is not statistically significant at the α = 0.05 level, the observed pattern aligns with previous reports that Gram-negative bacteria tend to have more open pangenomes, driven by larger accessory genomes and greater inter-strain gene content variability. Among Gram-positive taxa, *C. pseudotuberculosis* and *Lactococcus lactis* are outliers, exhibiting the lowest and highest gene discovery rates, respectively. Notably, *C*. *pseudotuberculosis* stands out as an extreme case of exceptionally limited gene discovery even in comparison to other Gram-positive taxa, reinforcing its characterization as a genomically stable species.

The contrast in gene discovery became even more pronounced when compared to highly diverse bacterial species such as *Escherichia coli*, *Salmonella* spp., and *Campylobacter* spp., which exhibited gene discovery rates approximately 4.5–6.5-fold higher than that of *C. pseudotuberculosis*. These findings highlight the species’ exceptionally clonal nature and limited genetic variability, consistent with previous reports describing its evolutionary stability.

#### Pan-genome characterization and biovar-specific gene content

**FIGURE 2:**
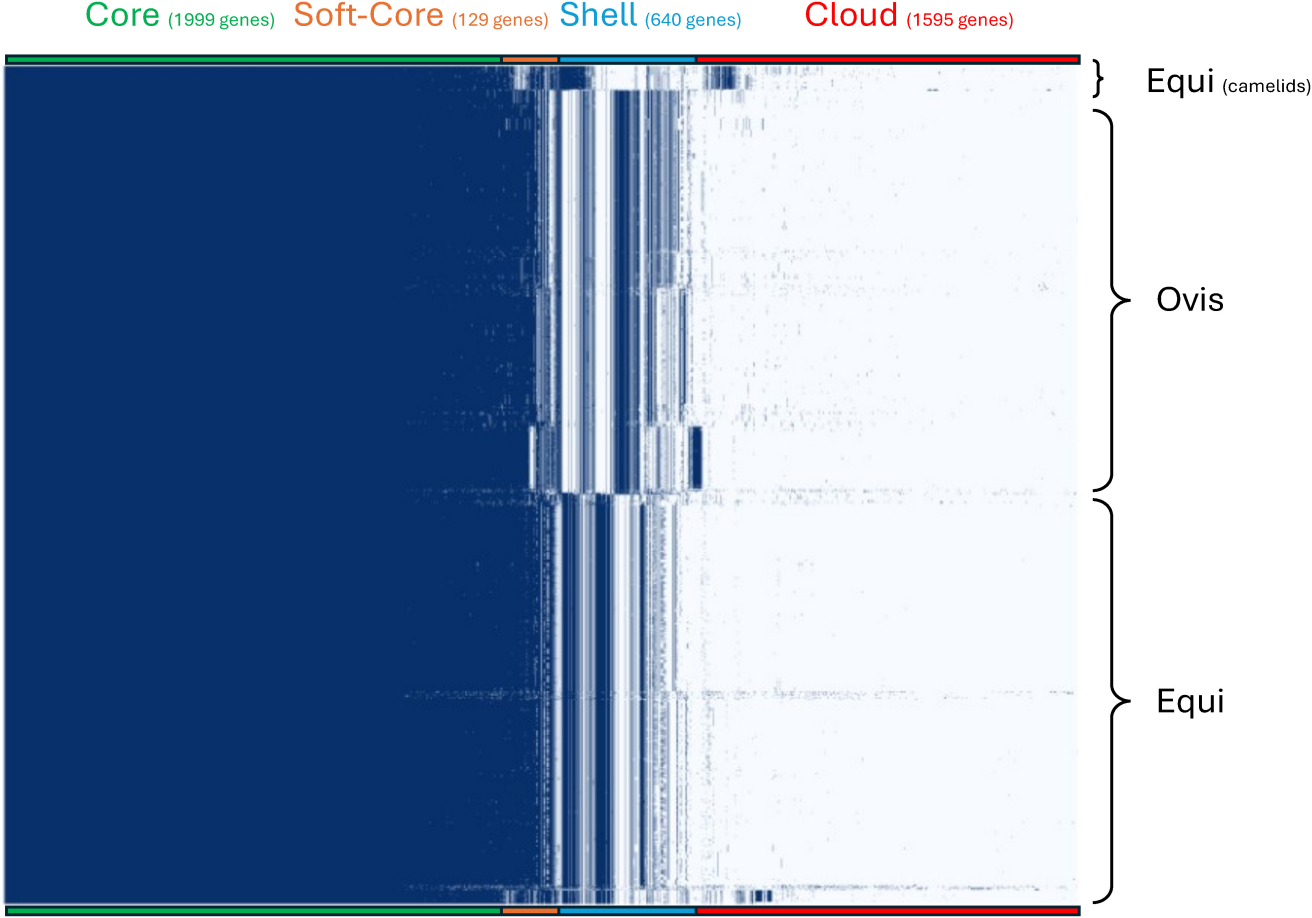
Gene presence-absence matrix of 788 Corynebacterium pseudotuberculosis genomes. The heatmap displays the distribution of 4,363 orthologous gene clusters across *C. pseudotuberculosis* genomes, clustered by presence (blue) or absence (white). Strains are grouped by biovar: “Equi” (408 genomes, top and bottom clusters) and “Ovis” (380 genomes, central cluster). A distinct subgroup within equi (top bracket) corresponds to camelid-derived isolates. Gene clusters (columns) are ordered and color-coded by frequency across the dataset: core (present in all strains, green; 1,999 genes), soft core (present in most strains, orange; 129 genes), shell (moderately distributed, light blue; 640 genes), and cloud (strain specific, red; 1,595 genes). The matrix reveals both shared and biovar-specific genomic content, highlighting differential gene retention patterns, particularly among accessory genes.

We performed a comprehensive pangenome analysis of the 788 *C. pseudotuberculosis* genomes to investigate the genomic architecture underlying biovar divergence and host adaptation. A total of 4,363 orthologous gene clusters were identified (Figure 2), which were categorized using presence-absence patterns into four major compartments: core (*n* = 1,999 genes), soft-core (*n* = 129), shell (*n* = 640), and cloud (*n* = 1,595). The core genome encompassed genes shared by all strains, while the accessory genome (soft-core, shell, and cloud genes) included elements with variable distribution across strains, many of which were biovar-specific. Notably, the clustering patterns revealed the same genomic signatures associated with the equi and ovis biovars as observed in the pairwise similarity analysis, including a distinct clade of equi strains primarily associated with camelid hosts (Figure 1).

**FIGURE 3:**
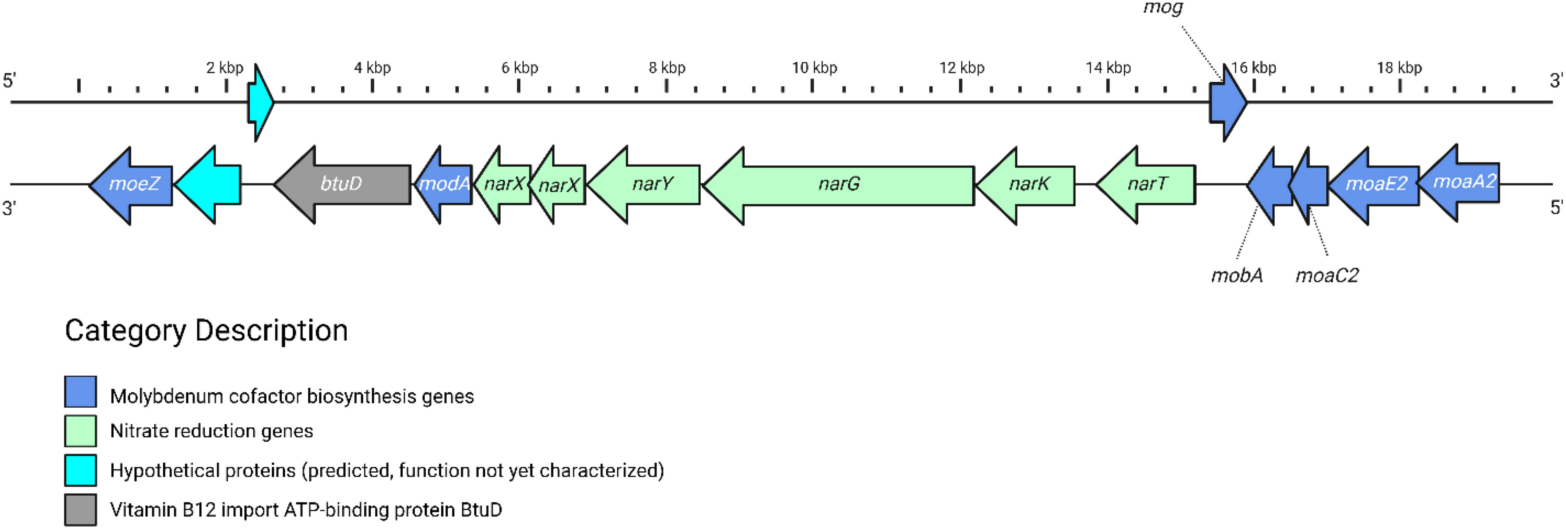
Organization of the *nar* Operon in Biovar equi. Syntenic arrangement of genes involved in nitrate reduction and molybdenum cofactor biosynthesis. Gene colors indicate functional categories: nitrate reduction (green), molybdenum cofactor biosynthesis (blue), hypothetical proteins (cyan), and a vitamin B12 import ATP binding protein (*btuD*) (gray).

While the core genome was highly conserved between ovis and equi isolates, a secondary level of conservation was observed within the shell genome, comprising genes present in biovar-specific strains, many of which appear to support distinct metabolic capabilities. In particular, biovar equi harbored a complete nitrate reduction operon (*narTKGYX*) and a molybdenum cofactor biosynthesis cluster (*moaA2, moaE2, moaC2, mobA, mog, modA*, and *moeZ*), which were absent in ovis isolates (Figure 3). This observation of biovar-specific genomic divergence in relation to nitrate-reduction is consistent with the nitrate-positive phenotype historically used to distinguish biovar equi.

While the capacity to reduce nitrate has been previously used as a biochemical test to distinguish biovars, a comparative presence-absence analysis revealed other metabolic functions unique to each biovar found in the shell genome. This analysis identified 159 genes exclusive to equi and 119 genes exclusive to ovis that aided in differentiating these biovars. Among these, 30 equi-specific and 15 ovis-specific genes were functionally annotated (Supplementary Tables 2, 3). Beyond nitrate metabolism, equi-specific genes revealed distinct metabolic and regulatory capabilities. These included genes involved in the transport and processing of amino acids and vitamins, such as *alsT* (amino acid carrier) and *btuD* (vitamin B12 import ATPase), as well as enzymes associated with polysaccharide metabolism, such as *treA* (trehalase). Genes related to oxidative stress response and energy production were also identified, including *guaB1* (putative oxidoreductase), *ppx1* (exopolyphosphatase), and *sdhL* (shikimate dehydrogenase-like protein). Additional genes linked to DNA recombination and genome plasticity, such as xer*C* (tyrosine recombinase) and *hin* (invertase), along with components of the CRISPR-Cas immune system (*casC*, *ygbT*), were also present. These findings suggest that biovar equi possesses broader adaptive potential in both host-associated and environmental contexts, beyond its well-characterized nitrate utilization pathways.

Ovis-specific genes encompassed a different set of metabolic and regulatory functions. Some were involved in amino acid and nitrogen metabolism, including *tdcG* (L-serine dehydratase), *ilvB1* (acetolactate synthase large subunit), *metXA* (homoserine O-acetyltransferase), and *pepN* (aminopeptidase N). Genes associated with transport systems included *acp* (sodium/proton-dependent alanine transporter), *bceA* (bacitracin export ATPase), *btuC* (vitamin B12 import permease), and *fecE* (ferric dicitrate transport ATPase). In addition, *rlmN* (putative RNA methyltransferase) and *recF* (DNA replication and repair protein) may play roles in regulatory control and genome maintenance. Other notable genes, such as *yidA* (a sugar phosphatase) and a fimbriae subunit, point to potential differences in environmental sensing and host interaction. Together, these biovar-specific accessory genes likely underpin distinct strategies for nutrient acquisition, redox balance, and surface adaptation, supporting their divergent ecological niches and host preferences.

One gene of particular interest among the biovar-specific sets was *fatD*, encoding a petrobactin import system permease protein. This protein is predicted to function as a membrane receptor for ferric-petrobactin, a catecholate-type siderophore with unique structural features that distinguish it from other bacterial iron-chelating compounds. Petrobactin is primarily produced by some *Bacillus* species such as *B. cereus*, *B. thuringiensis*, and *B. anthracis*. Remarkably, we identified two versions of *fatD* in C. *pseudotuberculosis*, one found exclusively in biovar equi and the other exclusively in biovar ovis. These allelic variants differ by six nucleotides, yet their translated amino acid sequences were identical, demonstrating complete conservation at the protein sequence level.

### Screening of virulence and antimicrobial resistance genes

In addition to metabolic capacity, virulence factors can also contribute to biovar-specific host adaptation. To assess the presence of virulence factors, genes that support a pathogen’s ability to infect a host and initiate disease, all 788 *C. pseudotuberculosis* genomes were analyzed using the Virulence Factor Database (VFDB). The phospholipase D gene (*pld*), which encodes a potent exotoxin, was found in all isolates, consistent with its known crucial role in *C. pseudotuberculosis* virulence. The diphtheria toxin gene (*tox*) was detected only in 11 isolates from buffalo hosts, in accordance with previous reports (Viana et al., 2017). No additional virulence-associated genes were identified by VFDB across the dataset, indicating that *pld* and *tox* represent the only detectable virulence factors under the applied screening criteria.

Subsequently, the genomes were screened for antimicrobial resistance (AMR) genes using multiple curated databases. While most genomes lacked significant matches to known AMR genes under standard thresholds, a subset of isolates carried resistance determinants. Specifically, the aminoglycoside resistance gene APH(3’)-IIa and the beta-lactamase gene TEM-116 were co-detected in seven isolates (*phoP*, SigmaE, *sigB, sigH, sigM, sigD, and sigC*), all derived from goat hosts in Brazil. In contrast, APH(3’)-IIIa was detected exclusively in a single, distinct isolate (Cp13), also from a goat in Brazil. All detected resistance genes exhibited high sequence identity (≥99.9%).

#### Core genome SNP analysis highlights clonality within biovars and limited geographic signal

Given the high degree of conservation of genes among the genomes, we next investigated whether differences between biovar ovis and biovar equi were associated with nucleotide mutation. Determining SNPs in the core genome using Snippy-core was done independently for each biovar. One high-quality representative genome for each biovar was used as a reference: strain 1002B (ovis; accession: GCF_001433475.1) and strain 258 (equi; accession: GCF_000263755.5). This analysis was restricted to isolates for which raw sequencing reads were available, allowing consistent read mapping, SNP calling, and quality filtering across samples.

Among the 330 equi isolates, 2,109 SNPs were identified in the core genome, while 241 ovis isolates displayed 1,206 SNPs. Per-genome SNP counts ranged from a few hundred to just over 2,000 in equi, and from under 1,000 to around 1,200 in ovis genomes, reflecting modest intra-biovar variability. Most isolates exhibited uniformly low SNP counts, reinforcing the clonal structure determine from gene-based analyses (Figures 1, 2).

**FIGURE 4:**
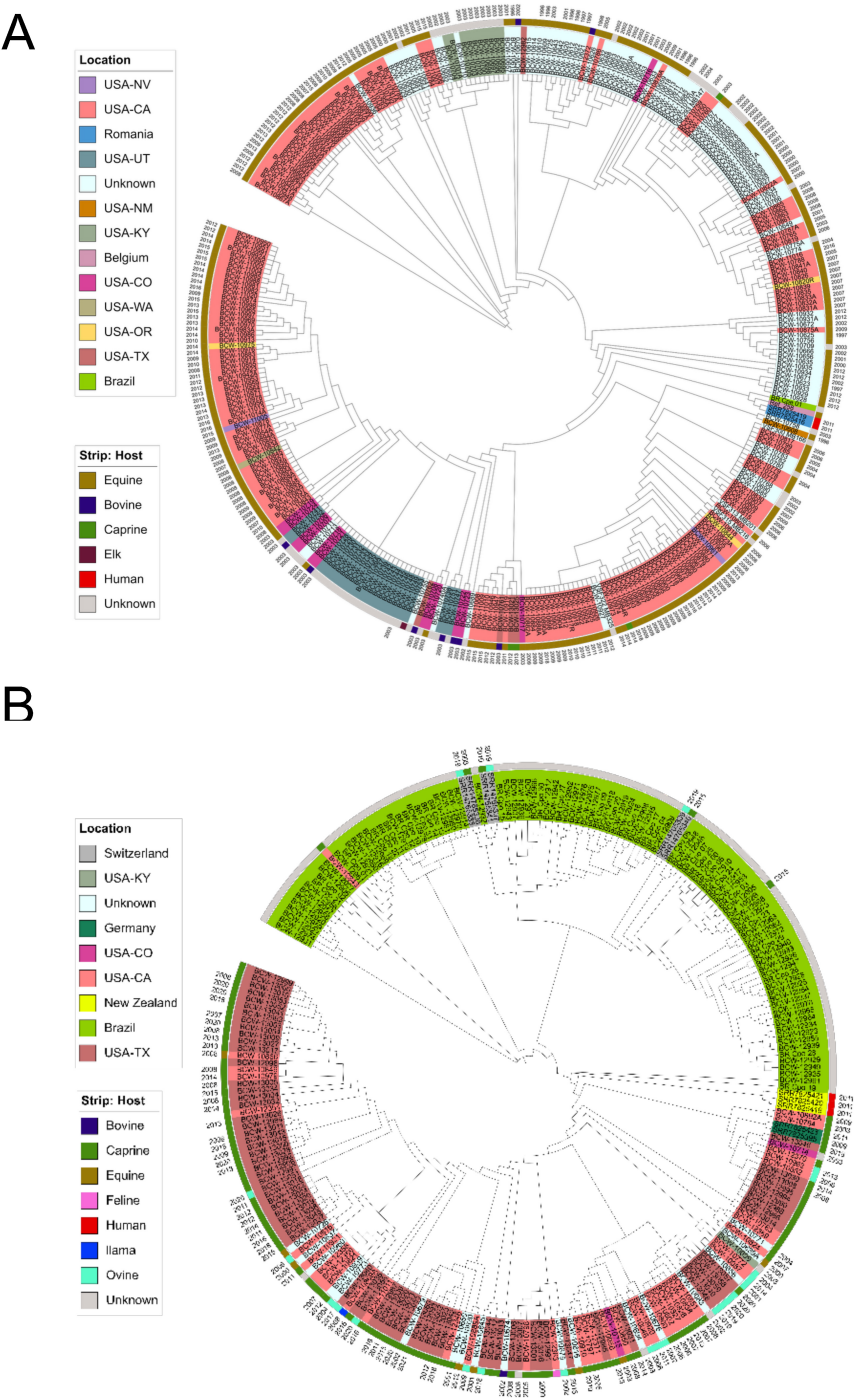
Core genome SNP phylogenies of *Corynebacterium pseudotuberculosis* biovars. **(A)** Phylogenetic tree of biovar equi constructed from core genome SNPs across 330 isolates. **(B)** Phylogenetic tree of biovar ovis constructed from core genome SNPs identified across 241 isolates. Trees were generated using alignments produced by Snippy core, representing high-confidence SNPs present in the conserved core genome of each biovar. Each tip is annotated with isolate ID and colored by geographic origin. Metadata rings indicate host species (inner strip) and year of isolation (outer ring). Colors correspond to the legends shown. Geographic origin is shown at the state level for U.S. isolates and at the country level for non-U.S. isolates based on metadata availability. In equi, no clear phylogenetic structure was observed based on host, location, or sampling date, consistent with a highly clonal population. In contrast, ovis displayed a modest geographic signal, with some regional clustering. These results suggest that biovar-specific traits are not strongly associated with core genome divergence.

To visualize SNP-related phylogenetic relationships and assess potential associations with host, geography, or temporal patterns, phylogenetic trees were constructed from the core SNPs (Figure 4). In equi, the resulting tree had no meaningful clustering by host species or sampling year (Figure 4A). This underscores the high genomic homogeneity within the biovar. Conversely, the ovis SNP tree (Figure 4B) exhibited a modest but noticeable geographic signal, with some clustering of isolates from New Zealand and Brazil. However, even in ovis, there was no clear association between SNP-defined clades and host species.

### Machine learning identifies SNPs associated with host and biovar differences

Building upon the observed high conservation of *C. pseudotuberculosis* genomes at both the genome content and SNP levels, machine learning was used to assess whether subtle patterns of nucleotide variation could distinguish isolates according to host species between biovars. The XGBoost algorithm was applied to the core genome SNP matrices with a total of nine host categories considered. Feature importance analysis revealed that classification was not driven by a single dominant variant but rather by the cumulative effect of multiple SNPs, each contributing modestly to model performance (Table 2). Feature importance scores reflect the relative contribution of each SNP to the model’s predictive accuracy during classification, rather than direct causal effects on host specificity.

**TABLE 2:** Important SNPs contributing to host species adaptation identified using XGBoost.

Rank	SNP description	Gene	Gene function	Importance
1	Missense_variant c.442G>A p.Val148Ile	*oppB1*	ABC-type dipeptide/oligopeptide/nickel transporter permease	0.0165
2	Missense_variant c.322G>A p.Ala108Thr	*rnhB*	Endonuclease RNase H, involved in RNA-DNA hybrid resolution	0.0041
3	Synonymous_variant c.613C>T p.Leu205Leu	*oppC4*	ABC-type dipeptide/oligopeptide/nickel transporter permease	0.0025
4	Frameshift_variant & stop_gained c.288_321dup	-	(Unannotated locus)	0.00098
5	Missense_variant c.166C>G p.Leu56Val	*bioD1*	Biotin biosynthesis protein (AAA domain)	0.00079
6	Synonymous_variant c.285C>T p.Gly95Gly	*ilvB*	Thiamine pyrophosphate-requiring enzymes acetolactate synthase pyruvate dehydrogenase (cytochrome) glyoxylate carboligase phosphonopyruvate decarboxylase	0.000247746
7	Missense_variant c.121A>G p.Ile41Val	*srtC1*	Sortase (surface protein transpeptidase)	0.000210489
8	Synonymous_variant c.315A>G p.Ala105Ala	*infB*	One of the essential components for the initiation of protein synthesis. Protects formylmethionyl-tRNA from spontaneous hydrolysis and promotes its binding to the 30S ribosomal subunits. Also involved in the hydrolysis of GTP during the formation of the 70S ribosomal complex	0.0001408
9	Missense_variant c.823G>A p.Ala275Thr	*dapC*	Aspartate tyrosine aromatic aminotransferase	0.000135756
10	Synonymous_variant c.684A>G p.Gly228Gly	*htaA*	Htaa	0.000133307
11	Missense_variant c.350C>A p.Ala117Glu	*yhgF*	Accessory protein	0.000120098
12	Synonymous_variant c.441G>A p.Gln147Gln	–	Transport system, ATP-binding protein	0.00011504
13	Missense_variant c.2990C>T p.Ala997Val	*smc*	Required for chromosome condensation and partitioning	0.00010732
14	Missense_variant c.374T>C p.Val125Ala	–	Belongs to the alpha-IPM synthase homocitrate synthase family	0.000104831

Importance refers to the improvement in accuracy attributed to splits using the SNP across all decision trees in the model.

The top-ranked SNP was a missense variant (c.442G>A; p.Val148Ile) within the *oppB1* gene, encoding a subunit of an ABC-type dipeptide/oligopeptide/nickel transporter. Additional important variants included a missense mutation in *rnhB* (an RNase H enzyme), a synonymous variant in *oppC4* (another oligopeptide transporter), and a missense variant in *bioD1* (involved in biotin biosynthesis) (Table 2). These SNPs were prioritized based on their importance within the trained model and collectively capture nucleotide patterns that improve discrimination among host-associated isolate groups. Individually, none of these variants uniquely defines a host species; instead, host classification emerges from the combined signal of multiple SNPs distributed across conserved metabolic and transport genes.

### Functional enrichment and network analysis of important SNPs reveal transport and amino acid biosynthesis as distinguishing processes

**FIGURE 5:**
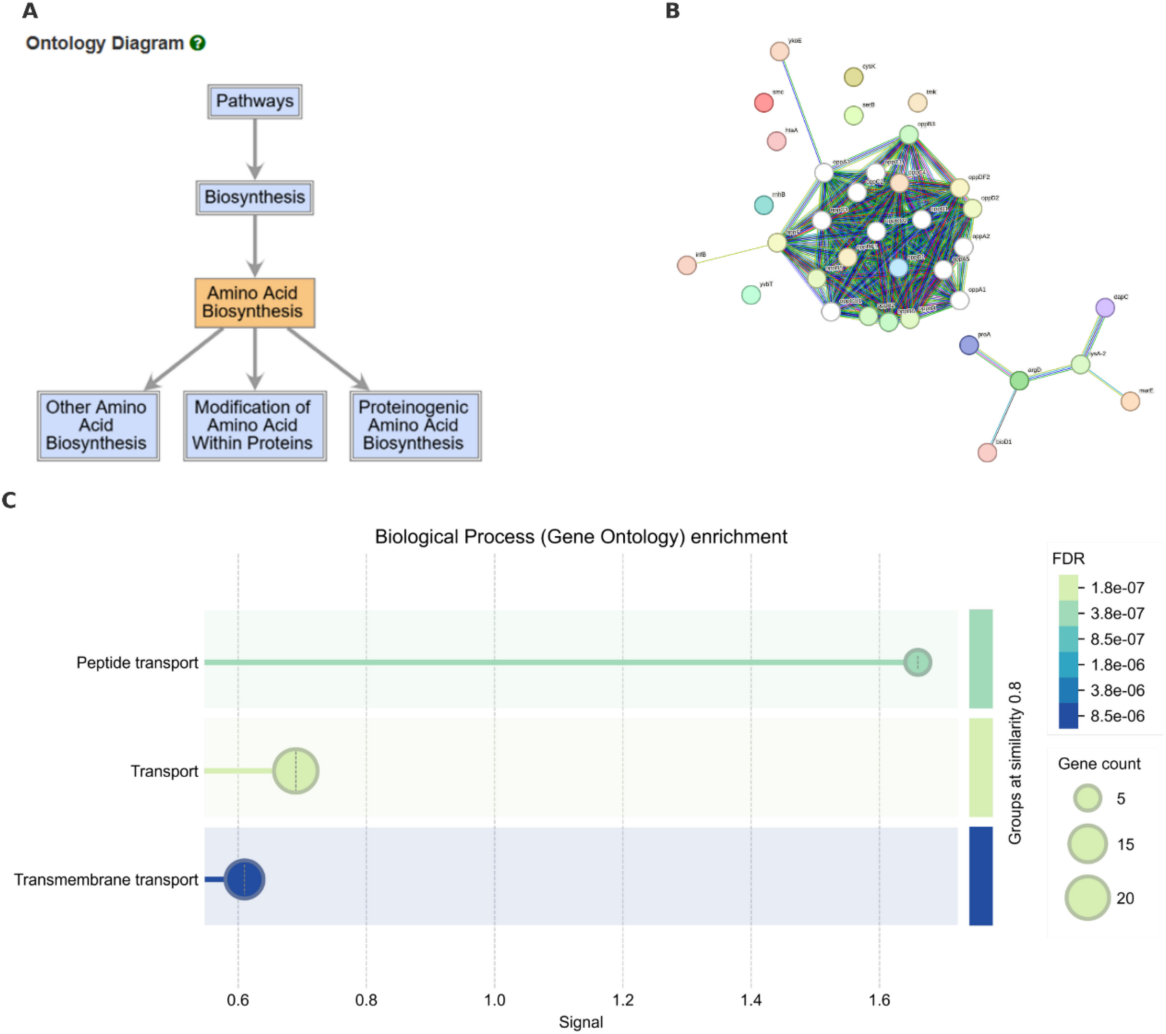
Functional enrichment and interaction network analysis of genes associated with host-specific SNPs. **(A)** BioCyc pathway ontology highlighting the enrichment of genes involved in “Amino Acid Biosynthesis” and related metabolic processes. **(B)** STRING-db protein–protein interaction network of top SNP-associated genes. A major cluster centered on Opp system transporters and a biosynthetic module including biotin and lysine biosynthesis enzymes are visible. **(C)** Gene Ontology (GO) enrichment graph displaying overrepresentation of biosynthetic pathways, particularly amino acid biosynthesis and protein modification processes. Circle size reflects the number of genes per GO term; color shading represents statistical significance (FDR).

Functional enrichment analysis of SNP-associated genes revealed a significant overrepresentation in the “Amino Acid Biosynthesis” ontology, which is a subclass of the broader “Biosynthesis” category (Figure 5A). Enrichment of amino acid processes suggests that a subset of core metabolic functions may be differentially modulated among isolates from different hosts.

Further analysis of protein-protein interactions for the gene products of top SNPs revealed two major functional modules (Figure 5B). The first comprised a densely interconnected cluster of oligopeptide permease (Opp) system components, including *oppB1*, *oppC4*, and *oppA*—*F* paralogs. The Opp system is a well-known ATP-binding cassette (ABC) transporter involved in peptide import and environmental sensing. This system has been associated to host adaptation in other bacterial pathogens, including *Streptococcus pneumoniae* and *Listeria monocytogenes* (Borezee et al., 2000). The second cluster was composed of biosynthetic enzymes, such as *bioD1* (biotin biosynthesis), *argD* (arginine biosynthesis), and *dapC* (lysine pathway).

GO enrichment (Figure 5C) further supported these findings. Statistically significant terms included “Amino Acid Biosynthetic Process,” “Proteinogenic Amino Acid Biosynthesis,” and “Modification of Amino Acids Within Proteins,” all with FDR values < 1.8 × 10^–6^. These pathways emphasize metabolic functions are associated with host-adaption in each biovar.

## Discussion

This study presents the most comprehensive population-scale genomic analysis of *C. pseudotuberculosis* to date, integrating 788 genomes across both biovars (ovis and equi). The results reveal a species of remarkable genomic stability, with highly conserved gene content and limited genetic variability despite broad host and geographic representation. The two biovars remain clearly distinguishable, yet both display near-clonal genome structures, indicating a species that evolves primarily through small-scale nucleotide changes rather than extensive horizontal gene transfer or gene gain/loss.

The pangenome analysis reinforces this view, showing a closed genome architecture and an exceptionally low gene discovery rate, the lowest reported among Gram-positive bacteria. For every ten newly sequenced genomes, only one additional gene was detected, underscoring the organism’s evolutionary rigidity. This extreme conservation contrasts with the open pangenomes of other pathogens such as *Escherichia coli*, *Salmonella* sp., and *Campylobacter* sp., where new genes emerge with nearly every additional genome. The high core-to-accessory gene ratio in *C. pseudotuberculosis* reflects a strong selective constraint, suggesting that its genetic repertoire is already well optimized for survival in its host niches.

Although accessory genome analyses confirmed classical biovar-specific features, such as nitrate reduction and molybdenum cofactor biosynthesis in equi, these gene differences alone do not explain host range or virulence variation. The uniform presence of the phospholipase D (*pld*) gene across all isolates supports its central role in virulence (Baird and Fontaine, 2007), while the detection of the tox gene exclusively in buffalo isolates aligns with previous findings (Viana et al., 2017). Beyond these canonical virulence determinants, no strong associations between gene presence/absence and host origin were observed, emphasizing that the determinants of host adaptation likely lie at the single-nucleotide level.

By integrating machine learning with SNP-based comparative genomics, we identified a set of subtle but informative allelic variants that discriminate isolates by host species. The top-ranked SNPs were found in genes linked to amino acid biosynthesis, peptide transport (Opp system), and cofactor metabolism, pathways central to nutrient acquisition and intracellular survival. These processes may facilitate fine-tuned metabolic compatibility between bacterial strains and host environments, consistent with the notion that *C. pseudotuberculosis* adapts through modulation of existing pathways rather than acquisition of new virulence genes.

This metabolic-centric model of adaptation is consistent with prior evidence implicating cell wall composition and enzymatic activity in *C. pseudotuberculosis* pathogenesis (Rebouças et al., 2020). The species’ thick, mycolic acid rich cell wall contributes to its resilience and cytotoxicity, while compositional differences in fatty acids and phospholipids have been linked to variation in virulence between isolates. Similarly, the activity of phospholipase D, which is universally present among strains, plays a dual role in tissue invasion and immune evasion. Together, these features suggest that *C. pseudotuberculosis* relies on fine scale metabolic and structural modulation rather than gene acquisition to navigate host defenses and persist in diverse environments.

The combination of genomic conservation, limited pangenomic expansion, and host-associated SNP signatures defines *C. pseudotuberculosis* as an unusually stable yet adaptable pathogen. Its evolution appears to proceed through incremental allelic shifts that optimize interactions within specific host environments, rather than through large-scale genomic innovation. This stability may explain the persistence of disease outbreaks over decades with minimal phenotypic change, despite global spread and host diversity.

## Conclusion

This work provides the most extensive comparative genomic analysis to date for *C. pseudotuberculosis*, integrating nearly 800 genomes spanning multiple hosts and global regions. The analyses revealed an organism with extraordinary genomic stability, characterized by a closed pangenome and minimal gene turnover. Such conservation contrasts with the dynamic gene exchange typical of other veterinary pathogens, positioning *C. pseudotuberculosis* as one of the most genetically homogeneous bacterial species examined at this scale. Gene content alone did not account for host adaptation or disease variation. Despite this stability, distinct biovar signatures persisted, including the nitrate reduction and molybdenum cofactor biosynthesis operons that define equi isolates. The lack of gene content differentiation let to the integration of machine learning with SNP-based comparative genomics that identified allelic variants within conserved metabolic pathways, particularly those involved in amino acid biosynthesis and peptide transport. These results indicate that *C. pseudotuberculosis* adapts through fine-scale genetic modulation of metabolic networks rather than through acquisition of classical virulence or resistance determinants. Collectively, these findings redefine *C. pseudotuberculosis* as a pathogen that evolves through precision rather than plasticity, maintaining a stable genetic core while fine-tuning key metabolic traits for host adaptation. This evolutionary strategy likely contributes to its persistence in livestock populations and its resilience across environments.

## Data Availability

The datasets presented in this study can be found in online repositories. The names of the repository/repositories and accession number(s) can be found at: https://www.ncbi.nlm.nih.gov/, PRJNA203445.
